# The nucleoside diphosphate kinase NDK-1/NME1 promotes phagocytosis in concert with DYN-1/Dynamin

**DOI:** 10.1096/fj.201900220R

**Published:** 2019-07-17

**Authors:** Zsolt Farkas, Metka Petric, Xianghua Liu, Floriane Herit, Éva Rajnavölgyi, Zsuzsa Szondy, Zsófia Budai, Tamás I. Orbán, Sára Sándor, Anil Mehta, Zsuzsa Bajtay, Tibor Kovács, Sung Yun Jung, Muhammed Afaq Shakir, Jun Qin, Zheng Zhou, Florence Niedergang, Mathieu Boissan, Krisztina Takács-Vellai

**Affiliations:** * Department of Biological Anthropology, Eötvös Loránd University, Budapest, Hungary;; † INSERM, Unité 1016, Institut Cochin, Paris, France;; ‡ Centre National de la Recherche Scientifique (CNRS), Unité Mixte de Recherche (UMR) 8104, Paris, France;; § Université Paris Descartes, Sorbonne Paris Cité, Paris, France;; ¶ Verna and Marrs McLean Department of Biochemistry and Molecular Biology, Baylor College of Medicine, Houston, Texas, USA;; ‖ Department of Immunology, University of Debrecen, Debrecen, Hungary;; # Department of Biochemistry and Molecular Biology, University of Debrecen, Debrecen, Hungary;; ** Institute of Enzymology, Research Centre for Natural Sciences, Hungarian Academy of Sciences, Budapest, Hungary;; †† Division of Medical Sciences, Ninewells Hospital Medical School, Dundee, United Kingdom;; ‡‡ Department of Immunology and MTA-ELTE Immunology Research Group, Eötvös Loránd University, Budapest, Hungary;; §§ Department of Genetics, Eötvös Loránd University, Budapest, Hungary;; ¶¶ Verna and Marrs McLean Department of Molecular and Cellular Biology, Baylor College of Medicine, One Baylor Plaza, Houston, Texas, USA;; ‖‖ Sorbonne Université, University Pierre and Marie Curie (UPMC) Paris 06, INSERM, Unité Mixte de Recherche (UMR) S938, Saint-Antoine Research Center, Paris, France; and; ## Assistance Publique–Hôpitaux de Paris (AP-HP), Hospital Tenon, Service de Biochimie et Hormonologie, Paris, France

**Keywords:** metastasis inhibitor, apoptotic clearance, phagosome formation, phagosome maturation, actin cup

## Abstract

Phagocytosis of various targets, such as apoptotic cells or opsonized pathogens, by macrophages is coordinated by a complex signaling network initiated by distinct phagocytic receptors. Despite the different initial signaling pathways, each pathway ends up regulating the actin cytoskeletal network, phagosome formation and closure, and phagosome maturation leading to degradation of the engulfed particle. Herein, we describe a new phagocytic function for the nucleoside diphosphate kinase 1 (NDK-1), the nematode counterpart of the first identified metastasis inhibitor NM23-H1 (nonmetastatic clone number 23) nonmetastatic clone number 23 or nonmetastatic isoform 1 (NME1). We reveal by coimmunoprecipitation, Duolink proximity ligation assay, and mass spectrometry that NDK-1/NME1 works in a complex with DYN-1/Dynamin (*Caenorhabditis elegans*/human homolog proteins), which is essential for engulfment and phagosome maturation. Time-lapse microscopy shows that NDK-1 is expressed on phagosomal surfaces during cell corpse clearance in the same time window as DYN-1. Silencing of NM23-M1 in mouse bone marrow–derived macrophages resulted in decreased phagocytosis of apoptotic thymocytes. In human macrophages, NM23-H1 and Dynamin are corecruited at sites of phagosome formation in F-actin–rich cups. In addition, NM23-H1 was required for efficient phagocytosis. Together, our data demonstrate that NDK-1/NME1 is an evolutionarily conserved element of successful phagocytosis.—Farkas, Z., Petric, M., Liu, X., Herit, F., Rajnavölgyi, É., Szondy, Z., Budai, Z., Orbán, T. I., Sándor, S., Mehta, A., Bajtay, Z., Kovács, T., Jung, S. Y., Afaq Shakir, M., Qin, J., Zhou, Z., Niedergang, F., Boissan, M., Takács-Vellai, K. The nucleoside diphosphate kinase NDK-1/NME1 promotes phagocytosis in concert with DYN-1/dynamin.

Macrophages are professional phagocytes that play a critical role in innate and acquired immunity because of their special ability to internalize and degrade both pathogens and apoptotic cells. Phagocytosis is initiated by the interaction of specific receptors on the surface of the phagocyte with ligands on the targeted particle. Although all phagocytosis requires actin polymerization, phagocytosis mediated through different receptors uses common and distinct mechanisms to regulate actin dynamics (1). Thus, DYN-1/Dynamin (*Caenorhabditis elegans*/human homolog proteins), an atypical GTPase, has been shown to participate in the uptake of apoptotic cells (2), zymozan, IgG-opsonized, and C3b_i_-opsonized particles, although each of them is taken up by different phagocytic receptors (3). In the engulfment phase, in which particles are rapidly encircled and internalized by the phagocyte, DYN-1 contributes to the membrane extensions necessary for pseudopod formation (2, 3) and to the scission of the phagosome (4). During corpse degradation, DYN-1 function is once again essential for phagosome maturation (2).

DYN-1/Dynamin was originally identified in the fly as a determining factor in endocytosis-mediated neurotransmitter uptake (5). In the fly, *awd* (abnormal wing discs) was found in multiple mutagenesis screens as a surprisingly exclusive genetic interacting partner for *dynamin* (6). AWD is a member of the nonmetastatic clone number 23 (NM23) protein family and is responsible for about 98% of the fly nucleoside diphosphate kinase (NDPK) activity. NDPKs are primarily known to ping-pong from phosphate to nucleoside diphosphates through a highly conserved phosphohistidine intermediate to generate their cognate nucleoside triphosphates, thus providing energy for different cellular processes (7). The best characterized member of NM23 (known as NME in the international nomenclature) protein family is NM23-H1 [nonmetastatic isoform 1 (NME1)] (8). To explain the exclusivity of the NDPK–Dynamin interaction, Boissan *et al.* (9) showed that the cytosolic NM23-H1 *Caenorhabditis* or NME1 and the mitochondrial NM23-H4/NME4 isoforms function in one complex with the cytosolic Dynamin at the plasma membrane clathrin-coated pits and with the mitochondrial DYN-1–like GTPase at the mitochondrial inner membrane, respectively. NDPKs fuel GTP locally to Dynamin superfamily GTPases in order to permit them to work with the highest thermodynamic efficiency during membrane remodeling (10–12).

Here, we show that a worm NDPK, NDK-1, which recently emerged as a new player in apoptotic cell elimination playing a role in the apoptotic cell internalization (13, 14), is also an essential factor promoting phagosome maturation through its direct interaction with DYN-1/Dynamin in the model organism *C*. *elegans*.

We also found by testing various phagocytosis models that the positive effect exerted on phagocytosis by NDK-1 is evolutionarily conserved because mouse and human NDPK homologs NM23-M1 and NM23-H1, respectively, are also factors promoting phagocytosis.

## MATERIALS AND METHODS

### *C. elegans* strains

*C. elegans* strains were maintained at 20°C on OP50 bacteria–seeded nematode growth media plates (15). Wild-type worms were *C. elegans* variant Bristol (N2). Strains used were TTV2 eluEx1[NDK-1::gfp; unc-119(+)];unc-119(ed3)III and TTV3 eluSi1[NDK-1::GFP + cb-unc-119(+)]III.

### Plasmid construction and generation of the transgenic array

The P*_ced-1_ndk-1::mCherry* plasmid was constructed by PCR amplifying the *ndk-1* genomic DNA between the start and the stop codon (13) and inserting it into the Sal1 and *Xma*I sites of a vector that carries the *ced-1* promoter (16) and the *mCherry* reporter cassette (unpublished results). Transgenic lines were generated by microinjection of P*_ced-1_dyn-1::gfp* and P*_ced-1_ndk-1::mCherry* together as previously described by Jin *et al*. (17). Plasmids were injected alongside the coinjection marker pUNC76 [*unc-76(+)*] into *unc-76(e911)* mutant adult hermaphrodites as previously described by Bloom *et al*. (18), with non-Unc animals being identified as transgenic animals.

### Duolink *in situ* proximity ligation assay

To detect the NDK-1/DYN-1 protein complex at single molecule resolution in *C. elegans* embryos, an *in situ* proximity ligation assay (PLA) was performed as previously described by Söderberg *et al*. (19) according to the manufacturer’s protocol (Olink Biosciences, Uppsala, Sweden).

Extracted *C. elegans* comma-stage embryos from strain TTV2 were freeze-cracked then fixed with acetone and incubated with primary antibodies: anti–green fluorescent protein (GFP) (Merck monoclonal antibody 3580, diluted to 1:500) and anti–DYN-1 (kindly provided by Z.Z., diluted to 1:200). Secondary antibodies tagged with short DNA oligonucleotides were added. Hybridization, ligation, amplification, and detection were realized according to the manufacturer’s protocol (Olink Biosciences). The PLA signal corresponds to the Cy3 fluorescence. Coverslips were analyzed on an inverted wide-field microscope. For human macrophages, cells grown on coverslips were fixed with cold methanol, incubated with primary antibodies, and processed as previously described.

### Coimmunoprecipitation

Co-immunoprecipitation (co-IP) was performed as previously described by Rocheleau *et al*. (20). Mixed-stage worms from the strains TTV3 and N2 were homogenized with Ultra-Turrax homogenizer (Ika, Staufen, Germany) in lysis buffer: 25 mM HEPES NaOH (pH 7.4), 150 mM NaCl, 1 mM DTT, 0.5% Triton X-100, 1 mM EDTA NaOH, and protease inhibitor cocktail tablet (Roche, Basel, Switzerland). Two micrograms of monoclonal anti-GFP antibody (3E6; Thermo Fisher Scientific, Waltham, MA, USA) was used to capture NDK-1::GFP from worm extracts. The adequate complexes were detected by immunoblotting using anti–DYN-1 polyclonal antibodies (provided by Z.Z., diluted to 1:1000). For detection, alkaline phosphatase–conjugated anti-rabbit secondary antibody (MilliporeSigma, Burlington, MA, USA) with nitro-blue tetrazolium and 5-bromo-4-chloro-3′-indolyphosphate substrate (1:50; MilliporeSigma) was applied.

### IP and mass spectrometry analysis

Mixed-stage *C. elegans* was suspended in 5 vol of NETN solution (50 mM Tris/pH 7.5, 150 mM NaCl, 1 mM EDTA, and 0.5% NP-40) and lysed using a bead beater homogenizer at 4°C for 1 min. Lysate was cleared by ultraspin (100 kg, 20 min, 4°C). Five micrograms of affinity purified pAb against DYN-1 (21) or the control antibodies (pAb against CED-2, affinity purified) (unpublished results) was added to the clear lysate and incubated at 4°C for 1 h.

The antibody and lysate mixture was cleared by the ultraspin (100 kg, 20 min, 4°C). Twenty microliters of protein A agarose beads was added and incubated for 1 h at 4°C. The beads were collected by 500 g spin and washed 3 times with NETN. The washed beads were loaded to 4–20% precast Novex Tris-Glycine gel (Thermo Fisher Scientific), and the gel was stopped when the dye front reached half-length. Gels were minimally stained with Coomassie Brilliant Blue (Thermo Fisher Scientific). The lane was subsequently cut into 4 pieces and subjected to in-solution digestion with 100 ng of trypsin overnight, extracted twice with 100% acetonitrile, and dried in a Savant Speed-Vac (Thermo Fisher Scientific). Peptides were then dissolved in 5% methanol and loaded onto a BioBasic C18 column (Thermo Fisher Scientific). Thermo-Finnigan LC/LC-ESI-LTQ (Thermo Fisher Scientific) was run in a data-dependent mode in which each sample was eluted in a 35-min, 0–80% acetonitrile gradient, and each full mass scan was followed by 15 tandem mass spectrometry scans of most abundant ions. Spectral data were then searched against target decoy *C. elegans* refSeq database [National Center for Biotechnology Information (NCBI), released September 2016, containing 28,105 entries] in Proteome Discoverer 1.4 interface (Thermo Fisher Scientific) with Mascot algorithm (Mascot 2.4; Matrix Science, London, United Kingdom). Assigned peptides were filtered with a 1% false discovery rate and subject to manual verifications. Variable modification of oxidation of methionine and protein N-terminal acetylation was allowed. The precursor mass tolerance was confined within 4 Da with a fragment mass tolerance of 0.5 Da and a maximum of 2 missed cleavages allowed. The mass spectrometry data have been deposited to the ProteomeXchange Consortium (*http://proteomecentral.proteomexchange.org*) *via* the Mass Spectrometry Interactive Virtual Environment repository (MassIVE; *http://massive.ucsd.edu*; MSV MSV000081868) with the dataset identifier PXD008559.

### Experimental animals

The experiments were carried out with 4-wk-old or 2–4-mo-old C57B6 mice. Mice were maintained in specific pathogen–free conditions in the Central Animal Facility, and all animal experiments were approved by the Animal Care and Use Committee of University of Debrecen.

### Bone marrow–derived macrophage cell culture

Bone marrow progenitors were obtained from the femurs of 2–4-mo-old mice lavaged with sterile physiologic saline. Cells were allowed to differentiate for 5 d in DMEM supplemented with 10% fetal bovine serum, 20% conditioned medium derived from L929 cells, 2 mM glutamine, 100 U/ml penicillin, and 100 mg/ml streptomycin at 37°C in 5% CO_2_. Nonadherent cells were washed away after 3 d.

### Cell culture of primary human monocyte–derived macrophages

Blood of healthy donors was used (Etablissement Français du Sang, Ile de France, Site Trinité, France) with the appropriate prior ethics approval as stated in the Etablissement Français du Sang and INSERM agreements 15/EFS/012 and 18/EFS/030, ensuring that all donors gave a written informed consent and providing anonymized samples. Primary human peripheral blood mononuclear cells were isolated by density-gradient sedimentation in Ficoll (GE Healthcare, Waukesha, WI, USA). Then, monocytes were selected by adhesion to dishes for 2 h at 37°C in fetal calf serum (FCS)–free medium [Roswell Park Memorial Institute (RPMI) 1640 medium supplemented with 100 U/ml penicillin 100 μg/ml streptomycin and 2 mM l-glutamine (Thermo Fisher Scientific)]. They were differentiated into macrophages for 8 d in adhesion medium supplemented with 10% FCS and 10 ng/ml recombinant human macrophage colony-stimulating factor (R&D Systems, Minneapolis, MN, USA) as in (22, 23).

### Small interfering RNA transfection of primary macrophages

Small interfering RNA (siRNA) transfection of human monocyte–derived macrophages (hMDMs) was as previously described by Marion *et al*. (24). Briefly, macrophages at d 4–5 were washed twice and kept in complete medium at 37°C. The siRNA solution was prepared in OptiMEM medium (GlutaMax supplemented; Thermo Fisher Scientific) containing Lipofectamine RNAiMax reagent (Thermo Fisher Scientific) and siRNA at a final concentration of 240 nM. Cells were incubated at 37°C for the indicated time. siRNA were 5′-GGCUGUAGGAAAUCUAGUU-3′ for NME1 and control siRNA 5′-CGUACGCGGAAUACUUCGA-3′ for Luciferase (Eurogentec, Liège, Belgium).

Five-day–matured bone marrow–derived macrophages (BMDMs) were transfected with On-Target*plus* Smartpool siRNA specific for mouse Nme1 and On-TargetPlus Nontargeting Control Pool (Dharmacon, Lafayette, CO, USA) using the Dharmafect 1 Transfection Reagent (Dharmacon) according to Dharmafect’s Transfection Protocol. At 48 h after transfection, cells were harvested for detecting the protein level of Nme1 of transfected BMDMs by Western blot analysis.

### Western blot analysis

BMDMs were homogenized in ice-cold lysis buffer containing 0.5% Triton X-100. Protein concentration of samples was diluted to 2 mg/ml, and then the samples were boiled with an equal volume of Laemmli buffer. Electrophoresis was performed in 15% SDS-polyacrylamide gel. Separated proteins were transferred to an Immobilion-P transfer membrane (MilliporeSigma) and were probed with anti-mouse Nm23 (Abcam, Cambridge, MA, USA) or β-actin antibodies (MilliporeSigma). Protein bands were visualized by Immobilon Western Chemiluminescent HRP substrate (MilliporeSigma).

### *In vitro* apoptotic cell phagocytosis

To determine the effect of Nme1 gene silencing on the phagocytosis of apoptotic thymocytes, at 48 h after transfection, an *in vitro* apoptotic cell phagocytosis assay was performed. To generate apoptotic thymocytes, thymi were collected from 4-wk-old C57B6 mice, and thymocytes were isolated and cultured for 24 h (10^7^ cells/ml) in RPMI 1640 medium supplemented with 2 mM glutamine, 100 U/ml penicillin, 100 mg/ml streptomycin, and 0.5 μM CellTracker Deep Red Dye (Thermo Fisher Scientific) in the absence of serum. Stained apoptotic thymocytes were added to the BMDMs in a 5:1 (apoptotic cells:macrophage) ratio for 1 h. After coculture, apoptotic cells were washed away, and macrophages were detached by trypsinization. The percentage of macrophages engulfing apoptotic cells was analyzed on a Becton Dickinson FACSCalibur (Becton Dickinson, San Diego, CA, USA).

### *In vitro* phagocytosis assays with hMDMs

Phagocytosis assays were performed with adherent cells plated on glass coverslips (25). For microscopy, RBC were washed in PBS1X and incubated with anti-RBC antibodies for 30 min at room temperature, then washed and resuspended in serum-free medium. Zymosan particles were washed in PBS1X and resuspended in serum-free medium. After internalization of the IgG-RBC for the indicated times, cells were fixed in 4% paraformaldehyde (MilliporeSigma) and 4% sucrose for 45 min at 4°C, and external RBC were labeled for 30 min with labeled F(ab′)_2_ anti-rabbit IgG Alexa Fluor 488 in PBS and FCS 2%. Cells were then permeabilized with 0.05% saponine before intracellular labeling with the indicated antibodies or fluorescent phalloidin in PBS and saponine 0.05% or FCS 2%.

To quantify phagocytosis, the number of internalized RBC per cell was counted in 50 cells randomly chosen on the coverslips (phagosomes are identified by combination of phase contrast and fluorescent images) corresponding to the phagocytic index. To quantify association, the number of external + internal RBC per cell was counted in 50 cells randomly chosen on the coverslips corresponding to the association index. The indexes obtained were divided by the index obtained for control cells and expressed as a percentage of control cells.

Quantification of F-actin or other proteins’ recruitment in the phagocytic cups was performed as previously described by Braun *et al*. (25). Briefly, quantification was performed on ImageJ 64-bit software (NIH) on a selected region in 1 section of a 16-bit stack. Primary fluorescence intensities through the phagocytic cup and in the cell cortex were measured and background corrected. Ratio values (enrichment indexes) were calculated by dividing the fluorescence intensities in the phagocytic cups by the fluorescence intensities in the cell cortex and plotted. Image acquisition was performed on an inverted wide-field microscope (DMI6000; Leica Microsystems, Buffalo Grove, IL, USA) with a ×100 (1.4 NA) objective and an Orca Flash4.0 (Hamamatsu Photonics, Shizuoka, Japan). *Z*-series of images were taken at 0.3-µm increments.

### Statistical analyses

Statistical analyses were performed using unpaired, 2-sided Student’s *t* test in Microsoft Excel software except for the phagocytosis assay of hMDMs, in which a 1-sided *t* test was used. The level of statistical significance was set at *P* < 0.05.

## RESULTS

### The NM23-H1/NME1 homolog NDK-1 physically interacts with DYN-1/Dynamin in the worm

Previously, we investigated defects of apoptosis in *ndk-1(ok314)–*null mutant worms (13), finding that embryos and germ cells defective for NDK-1 accumulated apoptotic cell corpses significantly in excess of wild-type worms. Around the dying germ cells, NDK-1::GFP expression was observed in the gonadal sheath cells that are specialized for engulfment and clearance of germ line corpses. We recently reported that *ndk-1* exhibits a genetic interaction with *dyn-1/dynamin*, whose role is well documented in apoptotic cell elimination (2, 13).

**Figure 1:**
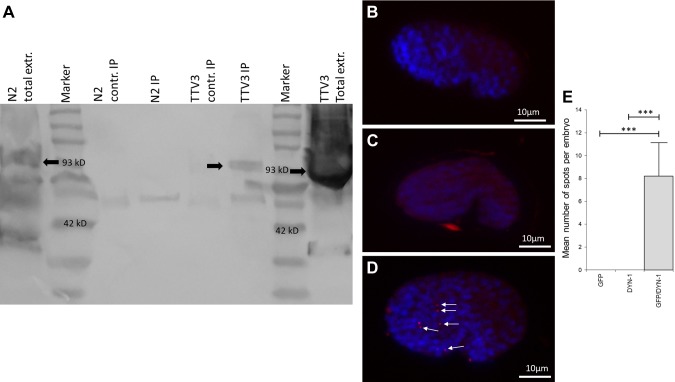
*A*) The NM23 homolog NDK-1 functions in a complex with DYN-1 in *C. elegan*. IP of NDK-1 with DYN-1 was performed by anti-GFP Ab (3E6) or by anti-tubulin mouse IgG as a control (contr.). Detection was done using anti-Dynamin. Arrows indicate DYN-1 (*C. elegans* homolog, 93 kD). Lanes: N2 (wild-type) total extract (extr.); MW marker; N2 (wild-type) extract precipitated by nonspecific (anti-tubulin) mouse IgG; N2 (wild-type) extract precipitated by mouse monoclonal anti-GFP antibody (3E6); TTV3 (NDK-1::GFP–transgenic line) extract precipitated by nonspecific (anti-tubulin) mouse IgG; TTV3 (NDK-1::GFP–transgenic line) extract precipitated by mouse monoclonal anti-GFP antibody (3E6); MW marker; TTV3 (NDK-1::GFP–transgenic line) total extract. *B*–*E*) DPLA shows that NDK-1 and DYN-1 are colocalized in *C. elegans* comma-stage embryos. Freeze-cracked and fixed embryos transgenic for NDK-1::GFP were stained by anti–DYN-1 and anti-GFP antibodies, and then the Duolink assay was performed. During microscopy, comma-stage embryos were selected (lateral view, anterior left). Blue staining (DAPI) indicates nuclei, and red dots (highlighted by arrows) represent Duolink signal. The Duolink assay only gives a positive signal if both species-specific antibodies are added to the sample. Labeling only with anti-GFP antibody (negative control I) (*B*). Labeling only with anti–DYN-1 antibody (negative control II) (*C*). Labeling with anti–DYN-1 and anti-GFP antibodies (*D*). Error bar represents sem. ***Significantly different from controls, determined by Student’s *t* test (*n* = 10) (*E*).

In order to further characterize the significance of the Dynamin/NDPK interaction in the worm during engulfment and corpse removal, we investigated the potential physical interaction between NDK-1 and DYN-1 by multiple approaches, including IP, IP–mass spectrometry, and Duolink PLA (DPLA). We found that DYN-1/Dynamin and NDK-1/NDPK co-immunoprecipitated in worms transgenic for NDK-1::GFP using a monoclonal anti-GFP antibody and probed with a highly specific polyclonal anti–DYN-1 antibody (21) (**Fig. 1*A***).

Furthermore, we performed an IP–mass spectrometry experiment in wild-type, mixed-stage worm extracts using the above-mentioned anti–DYN-1 antibody. A partial list of the proteins identified in the DYN-1 complex is presented in **Table 1**. The full mass spectrometry data are deposited in the ProteomeXchange Consortium. Dynamin was first identified as a microtubule-interacting protein (26). The presence of DYN-1, the original bait, and 3 tubulins (tubulins 1, 2, and 4), which are *C. elegans* microtubule components, validated that our experiments were properly conducted. In the same assay, we have also identified NDK-1 as a specific member of the DYN-1-complex but not of the control complex.

**TABLE 1:** Partial list of proteins coimmunoprecipitated with C. elegans DYN-1 and detected with mass spectrometry

Name	Control IP	DYN-1 IP	Peptides identified (*n*)	
71981891	Dynamin (*C. elegans*)	*dyn-1*	0	291
17552540	Tubulin β-2 chain (*C. elegans*)	*tbb-2*	0	191
17553980	Tubulin β-chain (*C. elegans*)	*tbb-1*	0	177
17549915	Tubulin β-4 chain (*C. elegans*)	*tbb-4*	0	90
17506807	Nucleoside diphosphate kinase (*C. elegans*)	*ndk-1*	0	11
193209657	Vitellogenin-2 (*C. elegans*)	*vit-2*	51	505
17570193	Vitellogenin-1 (*C. elegans)*	*vit-1*	32	339

Control IP are proteins pulled down using polyclonal antibodies against *C. elegans* CED-2.

During *C. elegans* development, 131 somatic cells are eliminated by apoptosis (27), and the vast majority (109) die during embyrogenesis (28). The dying cells are cleared by well-documented neighboring cells. This information enabled us to further analyze the colocalization of NDK-1 and DYN-1 *in vivo* through a DPLA performed on fixed *C. elegans* comma-stage embryos (Fig. 1*B–D*). In the comma stage, which is characteristic for mid embryogenesis, we previously reported a mean of 10.9 cell corpses per wild-type embryo (13). In the DPLA, which gives a positive signal only in case the antibodies recognizing the proteins of interest are in close proximity, we detected a mean of 8.2 positive signals per comma-stage embryo (Fig. 1*D, E*). This correlates well with the number of dying cells mentioned above (13) because apoptotic cells are eliminated by given neighboring cells. These data show that NDK-1 and DYN-1 function in the same complex in cells that have the potential to engulf apoptotic corpses in nematode embryos.

### Time-lapse microscopy shows that NDK-1 and DYN-1 are located on phagosomal surfaces at the early stage of phagosome maturation

**Figure 2:**
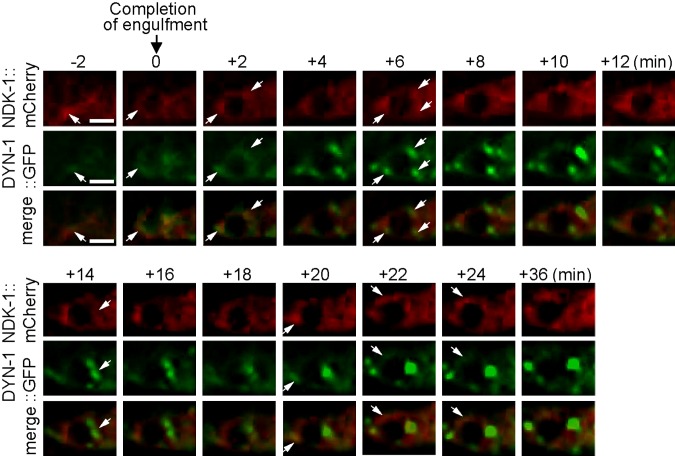
Both NDK-1 and DYN-1 are enriched on the surface of extending pseudopods and maturing phagosomes and partially overlap on the phagosomal surface. Time-lapse recording of DYN-1::GFP and NDK-1::mCherry, which are coexpressed in engulfing cells during the engulfment and degradation of cell corpse C3 in a wild-type embryo. “0 min” indicates the moment a nascent phagosome is just formed. Arrows indicate a few regions on extending pseudopods or the phagosome in which colocalization of GFP and mCherry is observed. Ten phagosomes were monitored by time-lapse recording, and the partial colocalization was observed from all of these phagosomes. Scale bar, 2.5 µm.

To further characterize the dynamics of NDK-1 and DYN-1 during apoptotic cell corpse removal in embryogenesis, we generated transgenic lines that coexpressed the DYN-1::GFP and NDK-1::mCherry reporters in engulfing cells under the control of the *ced-1* promoter. A time-lapse fluorescent microscopic imaging technique was used in living *C. elegans* embryos to monitor the engulfment and the subsequent phagosome maturation processes (29). Previously, DYN-1 was reported to be enriched on the surface of extending pseudopods and maturing phagosomes (2). Using this technique, we observed the enrichment of NDK-1::mCherry on the surface of a phagosome during the phagosome maturation process (**Fig. 2**). We further observed the partial colocalization of DYN-1::GFP and NDK-1::mCherry, in the punctate form, on the surface of a phagosome during the maturation process (Fig. 2). These results are consistent with cooperation of DYN-1 and NDK-1 during apoptotic clearance in a living organism.

### NM23-M1/NME1 is implicated in apoptotic cell engulfment and phagocytosis by mouse BMDMs

**Figure 3:**
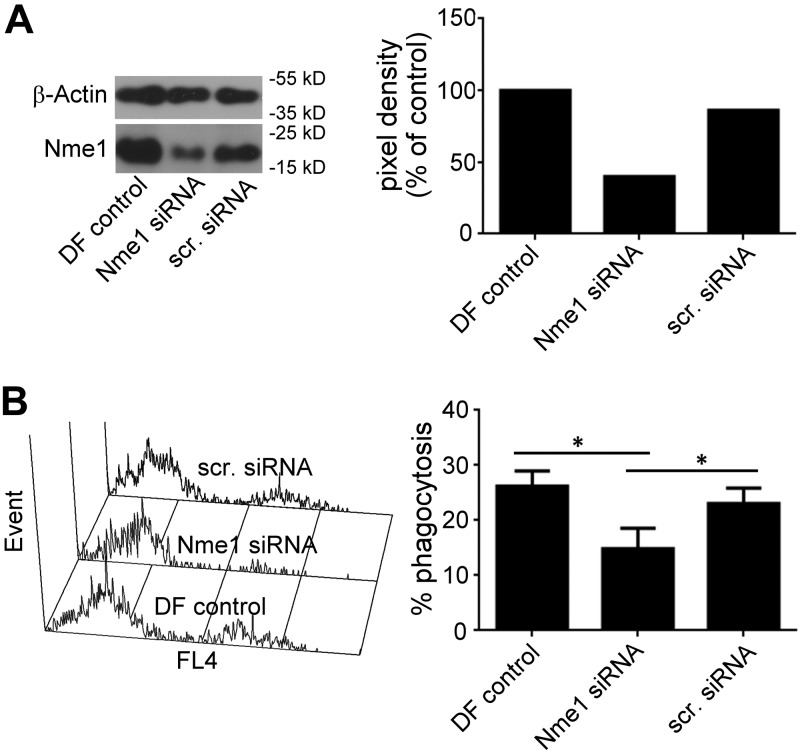
Silencing Nme1 significantly decreases the phagocytosis of apoptotic cells by mouse BMDMs. Macrophages were transfected with nontargeting siRNA or Nme1 siRNA by Dharmafect transfection reagent. At 48 h after transfection, cells were collected to determine protein levels of Nme1 by Western blot analysis (*A*) and to determine their phagocytic capacity (*B*) as described in Materials and Methods. Data represent the mean ± sd of 5 independent experiments. *Significantly different from respective control *P* < 0.05 determined by Student’s *t* test (*n* = 4).

Genetic pathways of apoptosis and apoptotic cell elimination are highly conserved between worms and mammals (14). Hence, we were interested to determine whether the involvement of NDK-1 in apoptotic cell engulfment is evolutionarily conserved. NM23-M1, the mouse homolog of NDK-1 was studied using BMDMs. Macrophages were treated by *Nm23-M1–*specific or nontargeting siRNAs and compared with reagent controls. Western blots showed that *Nm23-M1*–specific silencing resulted in a 55% decrease in NM23-M1 protein levels (**Fig. 3*A***). Next, an *in vitro* apoptotic cell phagocytosis assay was performed, in which apoptotic thymocytes were incubated with macrophages, and the phagocytic capacity of mouse macrophages was examined after *Nm23-M1* silencing. Decreased NM23-M1 level in specific or targeting siRNA-treated macrophages caused a 40% loss in their phagocytic activity (Fig. 3*B*).

### NM23-H1/NME1 is crucial to promote efficient phagosome formation in human macrophages

**Figure 4:**
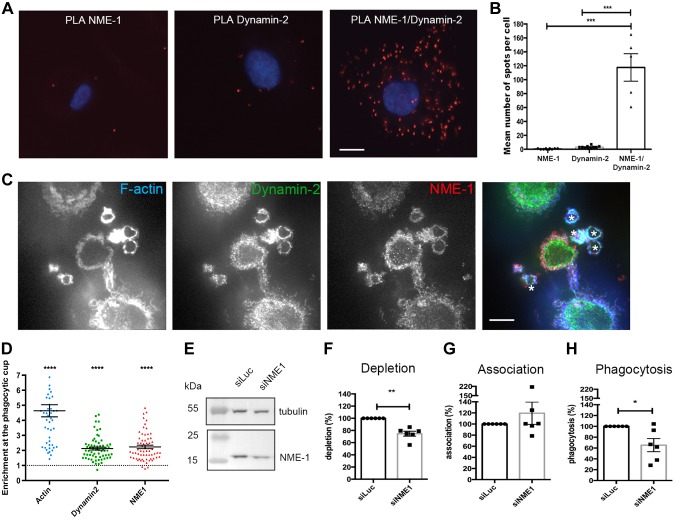
Role of NME1 in phagosome formation in human. Primary human macrophages were differentiated from blood monocytes. *A, B*) PLA was performed with anti-NME1 and anti-Dynamin antibodies. Images were acquired (*A*), and quantification of spots was performed and analyzed by ANOVA (*B*). Phagocytosis of zymosan was performed for 10 min before fixation, permeabilization, and labeling with phalloidin Alexa 635 and anti-NME1 followed by Cy3-coupled anti-mouse IgG antibodies and anti-Dynamin antibodies followed by Alexa 488–coupled anti-rabbit IgG antibodies. One confocal section is shown. *C*) Asterisks label the phagocytic cups. *D*) Protein enrichment in phagocytic cups was determined as described in Materials and Methods. *E–H*) Cells were treated with siRNA against NME1 (siNME1) or luciferase (siLuc) as a control for 72 h before cell lysis and Western blot analysis (*E*, *F*) or phagocytosis assay with IgG-opsonized red blood cells (*G*–*H*). Association (*G*) and phagocytosis (*H*) efficiencies were calculated as indicated in Materials and Methods and expressed as a percentage of control cells treated with siLuc for 6 different donors. Error bars represent sem. For depletion, association, and phagocytosis, we used 1-sided Student’s *t* tests. Scale bar, 5 µm. Error bars represent sem. *P < 0.05, **P < 0.01, ***P < 0.001, ****P < 0.0001.

Next, we analyzed the interaction of NM23-H1/NME1 and Dynamin in primary human macrophages derived *in vitro* from blood monocytes using DPLA (**Fig. 4*A, B***). The interaction was specifically detected in cells labeled with both the anti-NME1 and anti-Dynamin antibodies. The latter was a highly specific polyclonal anti-Dynamin antibody working in immunofluorescence and DPLA (9). To gain further insight into the role of NM23-H1/NME1 NME1 in human phagocytes, we examined the recruitment of endogenous NM23-H1/NME1 and Dynamin during phagocytosis of nonopsonized zymosan chosen to allow staining with specific antibodies against both proteins (Fig. 4*C*). Confocal sections revealed corecruitment of both NME1 and Dynamin in phagocytic cups, defined by the presence of F-actin (Fig. 4*C*). We calculated the enrichment of F-actin around the particles as compared with nonphagocytosing parts of the cell cortex and found that actin was enriched more than 4-fold at sites of phagocytosis (Fig. 4*D*). Similarly, NME1 and Dynamin were recruited in phagocytic cups with indexes of more than 2, showing clear enrichment at sites of phagocytosis in human macrophages of both NME1 and Dynamin.

To determine more precisely whether NM23-H1/NME1 plays a role in phagosome formation, we depleted NME1 by RNA interference (Fig. 4*E–H*). The depletion is never complete in macrophages (24), but there was a clear and significant reduction in the expression of NME1 in macrophages treated with a specific sequence (9) as compared with a control siRNA sequence (Fig. 4*E, F*). We then performed phagocytosis of IgG-opsonized particles on siRNA-treated cells and monitored the efficiency of initial particle binding (2 min, Fig. 4*G*) and internalization (60 min, Fig. 4*H*). The results obtained on cells from 6 donors revealed a significant impairment of phagocytosis in cells depleted from NME1 as compared with the control, whereas there was no significant modification of particle association to the cells. These results show that NME1 is important for an efficient receptor-mediated phagocytosis in human macrophages.

## DISCUSSION

Phagocytosis is the mechanism of internalization of large particles used to clear microorganisms but also to remove cell debris, which is important during development (4). The process of plasma membrane extension around the engulfed particle is driven by actin cytoskeleton rearrangement around the phagosome. It was recently shown that Dynamin is important for actin remodeling during phagosome formation and for phagosome closure (3, 4). The amount of GTP in cells is much lower than ATP and might be further limited in the membrane extensions. NME1 as an NDPK was shown to flux a high amount of GTP for optimized Dynamin GTPase activity during endocytosis (9).

However, the significance of the Dynamin–NDPK interaction and the exact mechanism by which NDPK contributes to apoptotic cell clearance were not yet known in any animal models. Therefore, in the present study, we expanded our previous data (13) on the relationship of NDK-1 and DYN-1 during apoptotic clearance in *C. elegans*, in which genetic pathways involved in apoptotic corpse removal are extensively characterized (14). It is well known that DYN-1/Dynamin is an essential component of the machinery involved in apoptotic cell removal through its membrane-remodeling activity (2) in both phases of apoptotic corpse removal, engulfment and elimination. We showed by IP and mass spectrometry that the 2 proteins physically interact in the nematode. Furthermore, the presence of the NDK-1/DYN-1 complex was also proven *in situ* by PLA in comma-stage embryos, which is a typical developmental stage in which apoptosis occurs. In addition, we found that NDK-1 also contributes to the process of phagosome maturation because it is expressed on phagosomal surfaces at the same time window of the elimination process as DYN-1. During this process, occasionally we observed colocalization of the 2 proteins, suggesting that NDK-1 might provide GTP locally to DYN-1/Dynamin. Thus, we report that NDK-1, as a potential GTP supplier for DYN-1, promotes both the engulfment and phagosome maturation phase of the apoptotic clearance process in the worm. DYN-1 is known to act downstream of the phagocytic receptor CED-1 in the clearance of apoptotic cells (2). Recently, the pathway led by CED-1 was reported to promote both the clearance of damaged axonal debris but also the regeneration of new axons by acting in muscle-type engulfing cells (30). The expression of *dyn-1* and *ndk-1* has been detected in body wall muscles (2, 31). We propose that NDK-1 and DYN-1 might also participate in these 2 new activities.

In *C. elegans* embryos, careful time-lapse recording and genetic analyses have demonstrated that the removal of apoptotic cells is a multistage process (32). Within phagosome maturation, the last step of dying cell clearance, there are also multistages, each of which is marked by a distinct set of protein and lipid molecules on phagosomal surfaces. In particular, upstream regulators, such as receptor CED-1, DYN-1, the small GTPase RAB-5, and the class-II PI3-kinase PIKI-1, are the first group of proteins to be recruited to the surface of nascent phagosomes, followed by the subsequent production of PtdIns(3)P and the enrichment of small GTPases RAB-2 and RAB-7 and the sorting nexins, which are the PtdIns(3)P effectors, to phagosomal surface (32). These sequential events eventually lead to the recruitment of early endosomes and lysosomes, the intracellular organelles that are essential for the degradation of apoptotic cells, to fuse to phagosomes (32). The recruitment of RAB-5, RAB-7, and PIKI-1 requires the phagosomal enrichment and the function of DYN-1, indicating that DYN-1 is an early factor (33, 34). The dynamic enrichment pattern of DYN-1 on nascent phagosomes is consistent with the stage in which its function is needed (2). Based on the above evidence, we hypothesize that DYN-1, as an upstream regulator, acts to prime phagosomal surfaces for the downstream events and dissociates from the phagosomal membrane once its job is done. As a positive regulator of DYN-1, NDK-1 is likely to follow the dynamic enrichment pattern of DYN-1 on the phagosomal surface.

We were interested to see whether NME1 plays a similar role during phagocytosis in mammalian cells and focused first on the Dynamin/NME1 interaction during internalization of phagocytic particles in primary human macrophages. We confirmed the presence of the Dynamin/NME1 complex in hMDM cells by the highly specific DPLA method. Localization studies of endogenous NME1 and Dynamin during phagocytosis of insert particles revealed corecruitment of the 2 proteins to the phagocytic cups. Next, phagocytosis of IgG-opsonized particles was used to monitor the effect of NME1 knockdown in human macrophages. Depletion of NME1 resulted in a significant impairment of phagocytosis.

Besides hMDMs, we also examined the function of the mouse NME1 homolog, NM23-M1, in mouse BMDMs during elimination of apoptotic thymocytes. Silencing of NM23-M1 in BMDM cells resulted in decreased phagocytosis of apoptotic thymocytes.

Importantly, we demonstrate that the phagocytosis-promoting function of the mammalian homologs NM23-M1 and NM23-H1/NME1 is evolutionarily conserved because silencing of the appropriate NME1 homologs caused a decreased phagocytic capacity in worms as well as in mouse BMDMs and hMDMs tested in various phagocytosis models. Thus, we propose NME1/Dynamin cooperation as a global mechanism of successful phagocytosis.

## Abbreviations

BMDM: bone marrow–derived macrophage
DPLA: Duolink PLA
FCS: fetal calf serum
GFP: green fluorescent protein
hMDM: human monocyte–derived macrophages
IP: immunoprecipitation
NDK-1: NDPK-like 1
NDPK: nucleoside diphosphate kinase
NM23: nonmetastatic clone number 23
NME1: nonmetastatic isoform 1
PLA: proximity ligation assay
RBC: red blood cell
siRNA: small interfering RNA

## References

[1] Allen L. A., Aderem A. (1996) Molecular definition of distinct cytoskeletal structures involved in complement- and Fc receptor-mediated phagocytosis in macrophages. J. Exp. Med. 184, 627–637 8760816 10.1084/jem.184.2.627 PMC2192718

[2] Yu X., Odera S., Chuang C.-H., Lu N., Zhou Z. (2006) C. elegans dynamin mediates the signaling of phagocytic receptor CED-1 for the engulfment and degradation of apoptotic cells. Dev. Cell 10, 743–757 16740477 10.1016/j.devcel.2006.04.007

[3] Gold E. S., Underhill D. M., Morrissette N. S., Guo J., McNiven M. A., Aderem A. (1999) Dynamin 2 is required for phagocytosis in macrophages. J. Exp. Med. 190, 1849–1856 10601359 10.1084/jem.190.12.1849 PMC2195719

[4] Marie-Anaïs F., Mazzolini J., Herit F., Niedergang F. (2016) Dynamin-actin cross talk contributes to phagosome formation and closure. Traffic 17, 487–499 26847957 10.1111/tra.12386

[5] Van der Bliek A. M., Meyerowitz E. M. (1991) Dynamin-like protein encoded by the Drosophila shibire gene associated with vesicular traffic. Nature 351, 411–414 1674590 10.1038/351411a0

[6] Krishnan K. S., Rikhy R., Rao S., Shivalkar M., Mosko M., Narayanan R., Etter P., Estes P. S., Ramaswami M. (2001) Nucleoside diphosphate kinase, a source of GTP, is required for dynamin-dependent synaptic vesicle recycling. Neuron 30, 197–210 11343655 10.1016/s0896-6273(01)00273-2

[7] Steeg P. S., Palmieri D., Ouatas T., Salerno M. (2003) Histidine kinases and histidine phosphorylated proteins in mammalian cell biology, signal transduction and cancer. Cancer Lett. 190, 1–12 12536071 10.1016/s0304-3835(02)00499-8

[8] Steeg P. S., Bevilacqua G., Kopper L., Thorgeirsson U. P., Talmadge J. E., Liotta L. A., Sobel M. E. (1988) Evidence for a novel gene associated with low tumor metastatic potential. J. Natl. Cancer Inst. 80, 200–204 3346912 10.1093/jnci/80.3.200

[9] Boissan M., Montagnac G., Shen Q., Griparic L., Guitton J., Romao M., Sauvonnet N., Lagache T., Lascu I., Raposo G., Desbourdes C., Schlattner U., Lacombe M.-L., Polo S., van der Bliek A. M., Roux A., Chavrier P. (2014) Membrane trafficking. Nucleoside diphosphate kinases fuel dynamin superfamily proteins with GTP for membrane remodeling. Science 344, 1510–1515 24970086 10.1126/science.1253768 PMC4601533

[10] Takei K., McPherson P. S., Schmid S. L., De Camilli P. (1995) Tubular membrane invaginations coated by dynamin rings are induced by GTP-gamma S in nerve terminals. Nature 374, 186–190 7877693 10.1038/374186a0

[11] Sweitzer S. M., Hinshaw J. E. (1998) Dynamin undergoes a GTP-dependent conformational change causing vesiculation. Cell 93, 1021–1029 9635431 10.1016/s0092-8674(00)81207-6

[12] Roux A., Uyhazi K., Frost A., De Camilli P. (2006) GTP-dependent twisting of dynamin implicates constriction and tension in membrane fission. Nature 441, 528–531 16648839 10.1038/nature04718

[13] Fancsalszky L., Monostori E., Farkas Z., Pourkarimi E., Masoudi N., Hargitai B., Bosnar M. H., Deželjin M., Zsákai A., Vellai T., Mehta A., Takács-Vellai K. (2014) NDK-1, the homolog of NM23-H1/H2 regulates cell migration and apoptotic engulfment in C. elegans. PLoS One 9, e92687 24658123 10.1371/journal.pone.0092687 PMC3962447

[14] Conradt B., Wu Y.-C., Xue D. (2016) Programmed cell death during Caenorhabditis elegans development. Genetics 203, 1533–1562 27516615 10.1534/genetics.115.186247 PMC4981262

[15] Brenner S. (1974) The genetics of Caenorhabditis elegans. Genetics 77, 71–94 4366476 10.1093/genetics/77.1.71 PMC1213120

[16] Zhou Z., Hartwieg E., Horvitz H. R. (2001) CED-1 is a transmembrane receptor that mediates cell corpse engulfment in C. elegans. Cell 104, 43–56 11163239 10.1016/s0092-8674(01)00190-8

[17] Jin Y. (1999) Transformation. In C. elegans, a Practical Approach (Hope I. A., ed.), pp. 69–96, Oxford University Press, Oxford, United Kingdom

[18] Bloom L., Horvitz H. R. (1997) The Caenorhabditis elegans gene unc-76 and its human homologs define a new gene family involved in axonal outgrowth and fasciculation. Proc. Natl. Acad. Sci. USA 94, 3414–3419 9096408 10.1073/pnas.94.7.3414 PMC20384

[19] Söderberg O., Gullberg M., Jarvius M., Ridderstråle K., Leuchowius K.-J., Jarvius J., Wester K., Hydbring P., Bahram F., Larsson L.-G., Landegren U. (2006) Direct observation of individual endogenous protein complexes in situ by proximity ligation. Nat. Methods 3, 995–1000 17072308 10.1038/nmeth947

[20] Rocheleau C. E., Yasuda J., Shin T. H., Lin R., Sawa H., Okano H., Priess J. R., Davis R. J., Mello C. C. (1999) WRM-1 activates the LIT-1 protein kinase to transduce anterior/posterior polarity signals in C. elegans. Cell 97, 717–726 10380924 10.1016/s0092-8674(00)80784-9

[21] He B., Yu X., Margolis M., Liu X., Leng X., Etzion Y., Zheng F., Lu N., Quiocho F. A., Danino D., Zhou Z. (2010) Live-cell imaging in Caenorhabditis elegans reveals the distinct roles of dynamin self-assembly and guanosine triphosphate hydrolysis in the removal of apoptotic cells. Mol. Biol. Cell 21, 610–629 20016007 10.1091/mbc.E09-05-0440 PMC2820425

[22] Dumas A., Lê-Bury G., Marie-Anaïs F., Herit F., Mazzolini J., Guilbert T., Bourdoncle P., Russell D. G., Benichou S., Zahraoui A., Niedergang F. (2015) The HIV-1 protein Vpr impairs phagosome maturation by controlling microtubule-dependent trafficking. J. Cell Biol. 211, 359–372 26504171 10.1083/jcb.201503124 PMC4621833

[23] Mazzolini J., Herit F., Bouchet J., Benmerah A., Benichou S., Niedergang F. (2010) Inhibition of phagocytosis in HIV-1-infected macrophages relies on Nef-dependent alteration of focal delivery of recycling compartments. Blood 115, 4226–4236 20299515 10.1182/blood-2009-12-259473

[24] Marion S., Mazzolini J., Herit F., Bourdoncle P., Kambou-Pene N., Hailfinger S., Sachse M., Ruland J., Benmerah A., Echard A., Thome M., Niedergang F. (2012) The NF-κB signaling protein Bcl10 regulates actin dynamics by controlling AP1 and OCRL-bearing vesicles. Dev. Cell 23, 954–967 23153494 10.1016/j.devcel.2012.09.021

[25] Braun V., Fraisier V., Raposo G., Hurbain I., Sibarita J.-B., Chavrier P., Galli T., Niedergang F. (2004) TI-VAMP/VAMP7 is required for optimal phagocytosis of opsonised particles in macrophages. EMBO J. 23, 4166–4176 15470500 10.1038/sj.emboj.7600427 PMC524391

[26] Shpetner H. S., Vallee R. B. (1989) Identification of dynamin, a novel mechanochemical enzyme that mediates interactions between microtubules. Cell 59, 421–432 2529977 10.1016/0092-8674(89)90027-5

[27] Sulston J. E., Horvitz H. R. (1977) Post-embryonic cell lineages of the nematode, Caenorhabditis elegans. Dev. Biol. 56, 110–156 838129 10.1016/0012-1606(77)90158-0

[28] Sulston J. E., Schierenberg E., White J. G., Thomson J. N. (1983) The embryonic cell lineage of the nematode Caenorhabditis elegans. Dev. Biol. 100, 64–119 6684600 10.1016/0012-1606(83)90201-4

[29] Li Z., Lu N., He X., Zhou Z. (2013) Monitoring the clearance of apoptotic and necrotic cells in the nematode Caenorhabditis elegans. Methods Mol. Biol. 1004, 183–202 23733578 10.1007/978-1-62703-383-1_14 PMC4038443

[30] Chiu H., Zou Y., Suzuki N., Hsieh Y.-W., Chuang C.-F., Wu Y.-C., Chang C. (2018) Engulfing cells promote neuronal regeneration and remove neuronal debris through distinct biochemical functions of CED-1. Nat. Commun. 9, 4842 30451835 10.1038/s41467-018-07291-x PMC6242819

[31] Masoudi N., Fancsalszky L., Pourkarimi E., Vellai T., Alexa A., Reményi A., Gartner A., Mehta A., Takács-Vellai K. (2013) The NM23-H1/H2 homolog NDK-1 is required for full activation of Ras signaling in C. elegans. Development 140, 3486–3495 23900546 10.1242/dev.094011 PMC3737725

[32] Lu N., Zhou Z. (2012) Membrane trafficking and phagosome maturation during the clearance of apoptotic cells. Int. Rev. Cell Mol. Biol. 293, 269–309 22251564 10.1016/B978-0-12-394304-0.00013-0 PMC3551535

[33] Yu X., Lu N., Zhou Z. (2008) Phagocytic receptor CED-1 initiates a signaling pathway for degrading engulfed apoptotic cells. PLoS Biol. 6, e61 18351800 10.1371/journal.pbio.0060061 PMC2267821

[34] Lu N., Shen Q., Mahoney T. R., Neukomm L. J., Wang Y., Zhou Z. (2012) Two PI 3-kinases and one PI 3-phosphatase together establish the cyclic waves of phagosomal PtdIns(3)P critical for the degradation of apoptotic cells. PLoS Biol. 10, e1001245 22272187 10.1371/journal.pbio.1001245 PMC3260314

